# The Life Course Implications of Ready to Use Therapeutic Food for Children in Low-Income Countries

**DOI:** 10.3390/ijerph14040403

**Published:** 2017-04-11

**Authors:** Alessandra N. Bazzano, Kaitlin S. Potts, Lydia A. Bazzano, John B. Mason

**Affiliations:** 1 Department of Global Community Health and Behavioral Sciences, Tulane University School of Public Health and Tropical Medicine, New Orleans, LA 70112, USA; kstorck@tulane.edu (K.S.P.); masonj@tulane.edu (J.B.M.); 2 Taylor Center for Social Innovation and Design Thinking, Tulane University, New Orleans, LA 70118, USA; 3 Department of Epidemiology, Tulane University School of Public Health and Tropical Medicine, New Orleans, LA 70112, USA; lbazzano@tulane.edu

**Keywords:** ready to use therapeutic food (RUTF), undernutrition, maternal, child, malnutrition

## Abstract

The development of ready-to-use therapeutic food (RUTF) for the treatment of uncomplicated cases of severe acute malnutrition in young children from 6 months to 5 years old has greatly improved survival through the ability to treat large numbers of malnourished children in the community setting rather than at health facilities during emergencies. This success has led to a surge in demand for RUTF in low income countries that are frequently food insecure due to environmental factors such as cyclical drought. Worldwide production capacity for the supply of RUTF has increased dramatically through the expansion and development of new manufacturing facilities in both low and high income countries, and new business ventures dedicated to ready-to-use foods have emerged not only for emergencies, but increasingly, for supplementing caloric intake of pregnant women and young children not experiencing acute undernutrition. Due to the lack of evidence on the long term health impact these products may have, in the midst of global nutrition transitions toward obesity and metabolic dysfunction, the increased use of manufactured, commercial products for treatment and prevention of undernutrition is of great concern. Using a framework built on the life course health development perspective, the current research presents several drawbacks and limitations of RUTF for nutrition of mothers and young children, especially in non-emergency situations. Recommendations follow for potential strategies to limit the use of these products to the treatment of acute undernutrition only, study the longer term health impacts of RUTF, prevent conflict of interests arising for social enterprises, and where possible, ensure that whole foods are supported for life-long health and nutrition, as well as environmental sustainability.

## 1. Introduction

Undernutrition in children worldwide is considered the direct or indirect cause of mortality in approximately 45% of all deaths among children younger than five [1], and has lifelong consequences for physical and cognitive wellbeing. In 2014, it was estimated that 50 million children under five globally were wasted (acutely malnourished), and 16 million of those were severely wasted, while 23.8% of children under five were stunted, representing a more chronic form of undernutrition [2].

Severe acute malnutrition (SAM), defined as <−3 z-scores of the median weight-for-length of the World Health Organization (WHO) growth standard, visible severe wasting, the presence of nutritional oedema, or mid-upper arm circumference of less than 115 millimeters (in children 6–59 months), can be treated through varying approaches [3,4,5,6,7]. Children suffering SAM with medical complications or those with no appetite need inpatient care with milk-based diets (e.g., “F-75” with 75 kcal/100 mL), which can be fed via nasogastric tube if needed [3], whereas uncomplicated cases, and children who have already been stabilized, can be rehabilitated at home or in the community with appropriate local or manufactured foods with a higher caloric value (100 kcal/100 mL) [8].

Ready-to-use therapeutic foods (RUTF) have become a common method of treating SAM and are easier to use and distribute during nutritional emergencies. Furthermore, RUTFs do not require water for preparation, and are shelf stable for up to 2 years, which are advantages over the former inpatient treatment, and are generally palatable to children with an appetite, as the usual form of RUTF is a sweet, peanut-based, nutrient-enriched paste [9]. These have proven effective at treating SAM and moderate acute malnutrition (MAM) in many settings [10,11,12,13,14,15], however, there is evidence that RUFT may not be effective when scaled up in primary health care settings [16]. The present review, referring primarily to non-emergency situations, considered the following: (1) important drawbacks and limitations to RUTFs; (2) increased demand for and use of these products; (3) investigation of the sustainability of RUTFs, both for long-term human health during global nutrition transitions, and environmental sustainability; and (4) strategies which may prevent or mitigate any future health issues arising from the use of RUTF in mothers and children.

### 1.1. History of Ready to Use Therapeutic Foods for Children

RUTFs have allowed for the treatment of uncomplicated cases of SAM with appetite in the home/community, with care provided by mothers and supervision by community health workers, commonly through the community-based management of acute malnutrition (CMAM) program which was officially endorsed by the World Health Organization and other international agencies in 2007 [8]. This approach has allowed for greater numbers of affected children to receive adequate treatment and reach full recovery [17]. Alongside this, the rapid success of the CMAM program has led to the use of RUTFs as the often preferred means of SAM treatment in preference to potentially more cost-effective and sustainable alternatives, as well as the development of similar products for use beyond the original intentions of RUTFs for the home-based treatment of SAM in emergencies. United Nations Children’s Fund (UNICEF) is the largest global procurer of RUTF and provides technical support and guidance to organizations on its use, but has also had to defend use of RUTF in a position paper [18] which noted that RUTFs should be seen as medical treatment for SAM and should not be viewed as a ‘panacea’ for treatment and prevention of all cases of malnutrition; they also noted care is needed to ensure that RUTFs do not become a replacement for nutritional best practices and consumption of normal household foods.

In treatment of SAM, once children have been stabilized and begin to have an appetite, they enter the rehabilitation or ‘catch up’ phase and can begin to have F-100 (100 kcal/100 mL) which requires clean water to reconstitute the formula [3,4]. Lack of clean water and refrigeration in communities and the need to measure and mix the product (increasing possibility of error), were the primary factors limiting the possibility of safe home-care for the majority of children suffering SAM. The advent of RUTFs overcame these practical problems. RUTFs were developed by various nutritionists and eventually, through the Institut de Recherche pour le Developpement (IRD), which blended the formula with peanut butter, produced a long-lasting, high-energy and high-protein therapeutic food providing 543 kcal/100 g (more than five times the energy density of F-100) and equivalent to F-100 in terms of nutrients/100 kcal [9,19]. The resulting product does not require additional resources by the end-user and is logistically attractive in emergency situations where large numbers have to be fed, local food is unavailable, or health care resources are strained. The first commercially produced RUTF was patented by the French producer, Nutriset, with IRD, and came to be known as Plumpy’Nut [20].

Nutriset’s patent of the process and method of production of its RUTF initially limited the possibility of local production in low-income settings where it was needed most. However, in 2005, Nutriset founded PlumpyField, a network of Plumpy’Nut manufacturers licensed to produce Plumpy’Nut through a franchise model [21]. Subsequently, many PlumpyField factories have been built in countries where the product is needed, and produce the RUTF from more locally sourced foods [21]. This expansion has led to a 7-fold increase in production capacity from 2007 to 2014, with Nutriset’s network now able to produce over 100,000 metric tons (MT) of product per year [22]. However, production capacity seems to be far outpacing actual production, perhaps with the aim to capture as much of the future market as possible and to improve ability to respond to emergencies; in 2014 53,000 MT of Nutriset products were produced [22].

Further, in 2010, the Nutriset patent was made available online (with an attendant usage agreement) for the benefit of developing countries, allowing independent producers to make peanut-based RUTF in selected countries [20]. It should be noted that this occurred only after a spate of adverse publicity including articles in the New York Times questioning ethics [23], as well as threats of litigation from other organizations hoping to produce the paste; though these threats ultimately failed to overturn the patent, Nutriset made its patent available for use in selected developing countries for the production of generic versions of RUTF. Through PlumpyField and the opening of the patent for use by developing countries, many companies are now producing RUTF, either as Plumpy’Nut (in the case of the PlumpyField network) or under another name, both in low-income country settings and in high-income settings.

Production of RUTF in the countries that need and use the product has many potential benefits. Locally-manufactured RUTFs using locally-sourced ingredients should perform as well as imported RUTFs (e.g., Plumpy’Nut) as long as these meet the WHO specifications for composition and adhere to careful quality control measures to avoid contamination of unprocessed peanuts with aflatoxin [24]. Alternative base ingredients that are grown in the country ensure the child does not become accustomed to foods that are normally unavailable. Local production would greatly improve sustainability, minimize transport of the end product and associated costs along with potential transport-related delays, reduce tariffs, and employ citizens in the countries where it is needed. Additionally, local production could theoretically boost the economy within these countries as long as the profits were reinvested locally and ultimately accrued to benefit residents.

Despite the shift towards increasing production in end-use countries, the large majority of Plumpy’Nut is still produced by Nutriset in France, with the next largest producer being Edesia (a Nutriset licensee) in the US—almost two-thirds of Plumpy’Nut produced in 2014 was manufactured by Nutriset (in France) with about one-quarter coming from the non-Edesia members of the PlumpyField network [22]. UNICEF has stated that 22% of their 2013 procurement came from Africa [25].

Since the initial development of Nutriset’s Plumpy’Nut, RUTF has become the preferred method of addressing nutritional emergencies through both the United Nations and non-governmental channels, and attendant resources have been channeled into the purchase and use of RUTF on an enormous scale. UNICEF’s demand for RUTF has increased from less than 9000 MT in 2009 to over 30,000 MT in 2014, with the large majority going to African countries such as Ethiopia, Kenya, and Somalia, where environmental conditions including cyclical drought cause re-occuring food shortages [26]. One thousand metric tons of RUTF treats approximately 76,500 children for about 3.6 million USD (based on an average price of $50 per 150-sachet carton at approximately 13.8 kg per carton) [27,28,29]. A UNICEF 2013 evaluation of CMAM found the purchase of RUTF accounted for 50% of CMAM operating costs in one Ethiopia case study [30].

The proliferation of RUTF for the treatment of SAM has led to an increase in producers and a parallel expansion of the product range for use in treating MAM, stunting and for meeting general nutritional needs as a form of prevention, rather than its initially intended purpose of treatment of severe acute undernutrition. It is likely that in many cases, this use of RUTF and these new products has by default resulted in replacement of some of the family foods children would normally be eating. It may also impact negatively on continued breastfeeding of children older than 6 months (children <6 months old are not given RUTF). It is costly and a heavy logistical burden for local health systems to import RUTF. Often, producers of RUTF products do not use locally sourced ingredients, nor does the majority of production occur in the end-use countries [22]. In short, currently RUTF is neither logistically sustainable nor financially feasible for the end users. The following sections provide evidence of these problems.

### 1.2. Expansion of Ready-To-Use Foods

In view of the wide success of RUTF, and specifically Plumpy’Nut, additional ready-to-use products have been developed and a new industry has evolved, producing ready-to-use foods for uses beyond therapeutic feeding for acute malnutrition. For example, in addition to the standard-bearer RUTF Plumpy’Nut, Nutriset, who explicitly uses the slogan “from treatment to prevention” [29], has introduced several new product categories based on the initial formula including: Plumpy’Sup (supplementary food for stunted children), Plumpy’Mum (supplementary food for pregnant and lactating women), Plumpy’Doz (micronutrient enriched supplementary food), Enuv’Mum and Enuv’Nutributter (micronutrient enriched foods for children and mothers with nutritionally adequate but low micronutrient value diets), in addition to other products. These new products are already being produced in very large quantities, with 22,000 of the 53,000 MT of Nutriset product produced in 2014 being products other than the original RUTF Plumpy’Nut [22].

Despite the disclaimers from Nutriset that these products should not replace nutritional best practices, the development, marketing, and use of such products as intended by the manufacturer could result in the replacement of nutritionally adequate family foods. Their use may also undermine the importance of providing nutrition counseling for improved infant and young child feeding and sustainable solutions for improving food availability and dietary diversity through, for example, improved agriculture. Self-confidence in child feeding behaviors for mothers and children may also be undermined.

### 1.3. Development of an Industry

The potential for further expansion of this product category, and its market worth, has not escaped the attention of the private sector and a large conflict of interest problem has developed. Privately financed factories producing RUTF can hardly keep up with the expanding demand for the products. Recently, Abbott Nutritionals, maker of infant formulas and breastmilk substitutes, built a production facility as a donation for the non-governmental organization Partners in Health to produce Nourimanba RUTF in Haiti [31]. Additionally, private entities putatively dedicated to improving nutrition worldwide (but having no vested interest in public health) have sprung up to produce RUTF and other ready-to-use products through business licenses with Nutriset PlumpyField or through other entrepreneurial ventures.

One such factory, owned and run by a self-described social enterprise called Edesia Nutrition, was started by a person who was inspired by an episode of 60 min wherein journalist Anderson Cooper proclaimed Plumpy’Nut to be a “miracle food” [32]. With no background or training in nutrition or public health, President of the Board of Trustees and Executive Director Navyn Salem (financed by her venture capitalist spouse, Vice President, Paul Salem), became a Nutriset licensee in 2009 and built a large factory in Rhode Island, United States to produce RUTF [23,33]; and the factory has just finished an expansion to double its production capacity in 2016.

By all accounts the company has thrived on its commercial production and sales of ready-to-use products, shipping 9000 MT per year, and advertises an ever expanding portfolio of products, including most of the Nutriset line along with their own proprietary product, Peanut POW, described as ‘a Lipid-based Nutrient Supplement Medium Quantity (LNS-MQ—50 g) for schoolchildren three years of age and older to complement foods available during school hours and to address nutrient gaps’ [34], which is marketed to afterschool programs and supplementary feeding programs in the United States. The Peanut POW packaging, in contrast to traditional Nutriset products, closely resembles that of a candy bar with a colorful blue, foil wrapper and animated peanut superhero. From the financial materials required by law to be provided publicly, it is stated that the product produced in largest quantities by Edesia is Plumpy’Sup, for supplementation and treatment of MAM, rather than Plumpy’Nut [35]. Edesia ostensibly operates as a non-profit, but website materials explicitly describe ‘securing new business opportunities related to commercial avenues in both domestic and international markets’, ‘executing annual sales plan to support revenue and profit objectives’, as well as ‘market research on target countries and regions’ and ‘establishing and growing company brand awareness to penetrate international markets with new or current products’ [36]. Notably, there are no such statements about improving the nutrition situation or public nutrition beyond the sales of RUTF. One portion of the website describes this: ‘We often tell visitors to our factory that one $55 box equals the life of one child’ [37]. Annual reports, blogs, and website and marketing materials of Edesia and other companies producing RUTF inevitably feature emotionally-targeted photos of undernourished (usually black) children from poor countries, photogenically clutching packages of RUTF, while others provide testimonials of mothers lauding the benefits of the product, and before and after photosets.

Similarly, MANA nutrition based in North Carolina with a factory that produces RUTF in Georgia is at work refining their own proprietary supplementary feeding product to prevent MAM [38]. Another company, Valid Nutrition, based in Ireland and operated as a not-for-profit (but which counts former Unilever executives on its board of directors) produces their own brand of RUTF at a factory in Malawi using large percentages of locally sourced ingredients. Valid has also already expanded its product lines beyond treatment of SAM to ready-to-use supplementary and complementary foods for treating MAM and preventing chronic malnutrition [39]. The 2015 annual report notes, ‘This is the first step in a progressive programme we have designed to start the process of unlocking this potentially massive market’ [40]. It recently expanded operations to India in partnership with India’s largest private food brand and dairy, Amul [40,41], which sells vast quantities of milk powder and sugar sweetened beverages, to manufacture RUTF there. The head of Valid recently stated, ‘We need evidence to demonstrate that a public/private collaboration selling complementary foods through retail channels can improve health whilst generating a financial return for investors’ [42]. The Valid Nutrition business model aims for the factories to be self-funding through more traditional business means of sales and marketing, rather than relying on government funding and charitable donations; the latest annual report states: ‘VALID Nutrition aims to be a self-sustaining social enterprise funded through the sales and marketing of effective low cost nutritional products’ [40]. Additionally, Valid Nutrition’s sister company, Valid International, which shares the same founder and executive director/chairman, acts as a consulting group, providing technical support on implementation, monitoring and evaluation of health and nutrition programs, including CMAM and chronic malnutrition listed as specific focus areas [43] (a quote from Valid International’s website indicates their role in providing technical support: “Valid International specialises in the provision of expert technical support and the research, development and implementation of techniques to improve the quality, impact and accountability of any endeavour that aims to improve global health and nutrition. Our mission is to improve global health and nutrition with evidence-based, equitable, high impact solutions”).

Where it happens, the practice of the physician (or his wife) owning the pharmacy next to the clinic, where prescriptions are filled, is regarded as highly undesirable due to the clear conflict of interest. NGOs whose mission is to provide technical assessments in emergencies should clearly have no role in then providing the supplies they may recommend, thus in the type of assistance favored.

These cases illustrate how expansion of producers of RUTF have led to a parallel expansion in the development of ready-to-use foods for consumption outside of the treatment of SAM in the form of ready-to-use supplementary and complementary foods for both treatment and prevention of moderate and chronic malnutrition in both children and women. It seems clear based on the language used by these companies themselves that the development of these products is more essential for their growth and prosperity than for the best interest of the targeted end user.

Other companies have sprung up capitalizing on the expanding demand for RUTF and specifically Plumpy’Nut, which is often referred to, without irony, as a “miracle food.” One private company called This Bar Saves Lives uses a buy-one-give-one model to provide one packet of Plumpy’Nut for each snack bar purchased and its website promotional video describes Plumpy’Nut this way: ‘look, this is a huge problem [malnutrition], but what’s crazy is there is a really simple solution’ [44,45]. This Bar Saves Lives advertises that they focus on both treatment, through the giving of PlumpyNut, and prevention, through the giving of Nutributter [46]. Similarly, Calorie Cloud, a partner of US-based RUTF producer MANA as well as UNICEF, publicizes the ability to help people in the US (notably corporate employees) ‘recycle’ their calories by counting activity and weight loss and donating equivalent RUTF packets through MANA [47]. In another example of private-public interest in RUTF, Toddler Food Partners, a US-based company which provides ‘knowledge, equipment and hands-on training to make Ready to Use Therapeutic Food (RUTF)’ has recently partnered in India with Hexagon Nutrition Private Limited (HNPL), a pharmaceutical corporation, General Mills India office, the State Nutrition Mission and Tata Chemicals among other Indian organizations, to study, scale up and produce a local ready-to-use food and has plans for the development of future products [48].

Similar to Edesia, these companies often employ arguably unethical marketing techniques that have been described as ‘social pornography’ in the past, using images of nearly naked African children in a most vulnerable state of extreme hunger and suffering, while showing little respect for the dignity of the person involved given the extremely personal nature of such a state [49,50]. The ‘Our Cause’ page of This Bar Saves Lives gives a perfect example of this, displaying a before and after illustration of an African boy who, in the ‘before’ image appears emaciated with ribs protruding, while looking at the camera with wide eyes and a frightened look; only in the ‘after’ picture does the boy wear a shirt [46]. The European NGO confederation for relief and development, CONCORD, has adopted a formal Code of Conduct on Images and Messages in which it describes that images should be used that respect human dignity, truthfully depict the broader context of the situation, and avoid stereotyping or sensationalizing, among other principles [51]. The Irish association of non-governmental development organizations, Dochas, published a visual guide to the Code [52], wherein several sections describe the types of imagery used by the companies described here (e.g., the image from This Bar Saves Lives) as examples of what not to do. The non-EU based companies identified in this paper may not be subject to this code, but the existence thereof within the international development community suggests an understanding of the unethical nature of such marketing practices.

This explosion of activity centered around production, marketing and sales of ready-to-use foods and corporate partnerships, amounts to the exploitation of undernutrition for revenue generation, and even in the instance of a not-for-profit organization, poses major ethical challenges to appropriate, objective use and promotion of RUTF for its intended purpose.

As Jeffrey Sachs wrote in response to news coverage distorting the use of RUTF, ‘of the billion or so people in our world suffering from undernourishment, Plumpy’Nut is appropriate only for a small fraction. Most of the chronically under-nourished need not a solution to acute under-nutrition through food aid but regular access to a long-term, balanced healthy diet’ [53]. Sachs, who warned that the general public should be helped to understand the differences between acute and chronic hunger, and the need for different approaches to address each, went on to describe the ‘real solutions of improved local agriculture, improved household dietary practices, and expanded access of the poor to basic healthcare’ as those needed to address global malnutrition.

UNICEF mentions, in its position paper on the use of RUTF, its firm intention to adhere to the established international norms and guidelines for infant and young child feeding [18]. These include: exclusive breastfeeding for the first six months of life, followed by continued breastfeeding and the use of appropriate complementary foods for children 6–24 months; micronutrient supplementation for vulnerable groups; and advocating best practices for child nutrition, health and hygiene. It is in the context of this important statement and ethical obligation that we argue the use of RUTF and related ready-to-use-foods in situations other than therapeutic treatment of child undernutrition does not constitute use of appropriate complementary foods nor best practices for child nutrition and health. The next sections outline the problems associated with RUTF.

## 2. Limitations of RUTF and Potential Impact on Life Course Health and Nutrition

RUTF are currently recommended only in children with Severe Acute Malnutrition and only for those with no medical complications and who have an appetite [8]. For other children, RUTF are not formally recommended, and even for children with SAM, the long term effects of RUTF are unclear.

### 2.1. Epigenetic and Metabolic Effects

RUTF and other ready-to-use foods may appear effective in the sort term, but cause disruption through programming of metabolic and physiological function throughout the lifespan. Epigenetics refers to mechanisms that lead to long-term changes in gene expression controlled by DNA modifications or alterations without changes in DNA sequence. The primary DNA sequence is generally fixed at conception, but epigenetic changes are dynamic and modifiable, probably throughout the life course.

Evidence suggests that both maternal overnutrition and undernutrition may promote obesity in offspring [54,55,56]. Evidence of impaired insulin secretion was seen at 4 years of age among small for gestational age babies who exhibited catch-up growth in childhood [57]. Children may go through repeated cycles of treatment with RUTF as they may meet criteria for treatment of undernutrition more than once [58]—relapse has been shown to be similar with treatment of RUTF versus a standard diet of flour porridge [59]. Increased consumption of RUTF and similar foods as part of a young child’s diet, whether through treatment for SAM, the expansion of ready-to-use foods as supplementary food for prevention of undernutrition, or modification of taste preference towards sweet foods and increased availability of high fat and sugary snacks, may result in permanent alteration of the epigenome and associated metabolic functions. This is in addition to challenges potentially introduced through in-utero programming in a group who are already at risk for obesogenic changes that may come along with the nutrition transition in adolescence and adulthood. Work done by Lussana et al. described famine exposure as being associated with a preference for fatty foods and a tendency to be less physically active when studied in men and women at 58 years of age [60]. Rapid weight gain in the early years of life has been associated with increased risk of obesity, excess adiposity, and metabolic syndrome in adulthood among children in high resource settings. However, few studies have been conducted in low resource settings, and those that have been conducted (such as that by Santos et al. 2015) do not examine the use of RUTF. [61,62,63,64,65].

The same countries that are receiving the most RUTF are those experiencing rapid changes in the nutrition landscape and increasing levels of obesity and chronic disease. The programs chosen to combat malnutrition must account for potential negative long-term effects across the lifespan that are relevant in the context of the nutrition transition and the double burden of over and undernutrition. Growth restriction in utero, rapid weight gain and undernutrition in childhood have shown some association with increased risk of obesity, diabetes, metabolic syndrome and associated chronic diseases in adulthood. Is it responsible to be introducing high-fat, high-sugar ‘nutrition’ supplements to these populations?

WHO recommends detailed steps on how caretakers should administer an appetite test and specific quantities (based on child weight) that should be consumed within 30 min to pass a test and be admitted into the OTP [66]. In some situations, WHO recommends against an appetite test: if a child has any general danger signs, pneumonia, persistent diarrhea, dysentery, measles, or malaria, or if RUTF is not available for an appetite test. In these cases children should be referred for inpatient therapy [66]. Besides the child weight-based amounts provided by WHO which varies from 1/8 of a packet of RUTF to ¾ of the packet, other training materials have provided different reference amounts of RUTF to be consumed for a child to pass the appetite test. One UNICEF guideline on CMAM described “return of appetite” as the ability to consume 75% of RUTF, though the requirement to enter the OTP was only that the child eat at least two small spoonfuls [67]. Training materials produced by FANTA mentioned that ‘the child should eat at least one-third of the packet or three teaspoons from a pot of RUTF to pass the test’ [68].

Determination of whether or not the child has passed the appetite test, or has a return of appetite, might be difficult to assess in practice. Assessing whether or not each child has passed the appetite test could be difficult in hectic scenes at outpatient feeding centers, especially given the ambiguity of this determination. The need for an appetite test is essential to ensure medically complicated cases that need special care are not missed and dangerously provided with RUTF instead of transfer to a health facility. Mothers and caregivers who administer the appetite test may not realize the comorbidities of their child and these may not be treated appropriately once the child enters the OTP.

### 2.2. Potential to Influence Young Children’s Taste Preferences

Another, more long-term drawback to the use of RUTFs is the potential to shape young children’s taste preferences. Historically, it has been demonstrated that young children from an early age prefer sweet tastes, and this is thought to be related to the consumption of breastmilk [69,70]. Exposure of young children to a single food, e.g., RUTF, which is sweet and has a fatty consistency, could be detrimental to future preferences and eating habits [71]. Exposure to foods and food choice behaviors prior to the age of 4 influence the acquisition of ‘food repertoire’, ultimately impacting dietary patterns over the lifespan [72]. Similarly, children may develop a resistance to healthy foods in the form of a ‘food neophobia’ when not exposed to a reasonable variety of foods with different flavors in childhood [73]. Inclinations for specific flavors are likely to develop early, through milk-related taste exposures. When mothers breastfeed, children are more likely to acquire a taste for a variety of foods, as breast milk composition will change slightly. At the point of introduction of solid foods, however, food preferences consolidate more quickly and develop based on repeated exposures to foods. The persistence of these early influences has not been fully elucidated [74], but it is exposures to a single food or product that could certainly alter the liking for new food in children [75]. As children have increased exposure to an unfamiliar food, they naturally increase the liking of that specific food, and along with that liking, their willingness to consume it [76,77,78]. The long term consequences of repeated exposure of young children with malleable taste preferences to a sweet, fatty food product that arrives in a foil package are likely to be negative and at minimum to induce resistance to family foods or other locally available foods that do not possess similar flavor characteristics.

Another long-term concern about the excessive use of RUTFs is their potential to alter consumption patterns. While bland foods are unlikely to be consumed in larger than necessary quantities for nutrition, palatable foods are often consumed in excess of energy requirements [79,80,81,82,83]. Neural pathways that enhance mood and activate reward systems are associated with pleasant and appealing foods [84]. ‘The striatum, insula, anterior cingulate cortex and midbrain regions including the ventral tegmental area and substantia nigra are active in representing reward in response to food’ [85]. The dopamine pathway is implicated in the reward response to consumption of delicious foods [86]. In addition, the orbito-frontal cortex encodes specific types of reward stimuli including various aspects of food: odor; visual input; temperature, viscosity, astringency and fat texture; and taste [87,88,89,90,91]. These aspects of food habituation do not bode well for the potential for lifetime consumption of sugary foods, packaged snack foods, and other unhealthful foods in the midst of the increasing problem of obesity in low-income settings.

### 2.3. Impact on the Gut Microbiome

The human gut hosts a vast array of microbes collectively known as the microbiome [92,93]. The cross-sectional relationships of the gut microbiota with obesity [94,95,96,97,98], type 2 diabetes [97,99,100,101,102,103,104,105] and dyslipidemia [99,106] have already been documented. Evidence of an etiologic role of the microbiota on metabolic risk has primarily been derived from animal experiments [107,108,109] and one small but promising clinical trial [110]. There is also evidence that the gut microbiome influences host eating behavior and is easily impacted by dietary changes: ‘Microbes in the gastrointestinal tract are under selective pressure to manipulate host eating behavior to increase their fitness, sometimes at the expense of host fitness. Microbes may do this through two potential strategies: (i) generating cravings for foods that they specialize on or foods that suppress their competitors… microbiota are easily manipulatable by dietary changes’ [111]. Research has also shown that the gut mocriobiome can contribute to low levels of inflammation, fatty acid tissue composition and energy production [112,113,114].

Additionally, undernutrition research in Malawian twins has implicated the gut microbiome as a causal factor of kwashiorkor [115], and there is evidence of disbiosis in the gut microbiome of kwashiorkor children. When researchers transferred the gut microbiota of kwashiorkor children into mice, evidence presented that the kwashiorkor gut microbiota quickly adapted to treatment with RUTF, but not full recovery, such that when the mice with kwashiorkor microbiome transitioned to the normal diet, they lost weight, implying more treatment is needed to fully recover from episodes of SAM with kwashiorkor than treatment with RUTF alone. The research was further interpreted to imply ‘therapeutic food alone is insufficient to induce a sustained shift in the gut ecosystem state… that would cause an undernourished individual to achieve a more healthy nutritional state…. Correction of this self-propagating condition will require multiple, concurrent measures to remold the environmental landscape of the host gut, thereby inducing a shift toward a gut microbiome with more efficient nutrient utilization (energy harvest) and fewer intrinsic proinflammatory properties’ [116]. The potential long-term impact of consumption of RUTF on the gut microbiome by children with SAM is not yet fully understood. The mass treatment of SAM children with a product that may be impacting their ability to sustain full recovery could prove irresponsible.

### 2.4. Replacement for Long-Term Nutrition and Health Support, Counseling and Services

Increased use of ready-to-use and “specially formulated foods” will result in changing nutrition behaviors and nutritional support provided to families in a negative direction, that is, away from whole foods, local foods and integration of enriched child food with normal family foods. It is also likely that providing specially formulated foods or ready-to-use foods, will mean that the distribution of these products will be made in lieu of traditional nutrition counseling and support services. Giving a product (a magic bullet) rather than enabling, supportive counseling on nutrition, linked/coordinated with services available locally, is not responsible public health practice.

Rather than promoting and providing industrially packaged (even if locally produced) foil-wrapped foods, nutrition organizations should be putting resources into sustainable and long-term strategies for improving healthy foods locally, providing nutrition counseling, and improving nutrition counseling programming that is not working well [117].

### 2.5. Replacing the Alternatives of Rehabilitation with Home Prepared Proridges or Enhanced Family Foods

An early documentation of therapeutic feeding by Mason et al. in 1974, described children transitioning from the milk-based diet of the stabilization phase to enriched family foods for rehabilitation, consisting of a ‘super-wot’ in the Ethiopian context—oil and protein enhanced wot (sauce) with injera (bread) to very good effect [118]. In 1994 Khanum tested the ability of children with protein energy malnutrition to recover at home after a short period in a rehabilitation center in Dhaka and found outcomes similar to inpatients and those who attended day care centers [119]. The group who rehabilitated at home did so with regular family foods, and this method was found to be the most cost effective option. Greco et al. found improved success in recovery of severely malnourished children by using an enhanced, locally available and acceptable porridge in addition to F100 in Uganda in 2002, compared to treatment with F100 milk alone, and found this option much cheaper than the burgeoning RUTF spreads [120]. Researchers in India found treatment of children with SAM with a diet of local, energy dense foods was suitable for rehabilitation in a retrospective study analyzing inpatient cases from 2001 to 2005 [121]. A 2013 Cochrane review compared treatment of SAM at home with RUTF to the standard treatment. The standard treatment included inpatient stabilization (typically with F100) followed by home treatment with specially formulated porridges (maize/soy flour) that could be prepared at home and were acceptable for the entire family, the flour was either fortified with the necessary vitamin/mineral needs or a supplement was given additionally. The reviewers found little evidence of conclusive differences in outcomes (including recovery, relapse, mortality and weight gain), concluding that either treatment method could be used to treat children with SAM in the community, depending on the context [59].

In a WHO 2006 technical paper evaluating the efficacy and effectiveness of community-based treatment of malnutrition, researchers concluded, ‘if carers can make energy- and protein-dense food mixtures at home, then domiciliary care is probably the best delivery system for community-based care. If carers cannot make such foods, then provision of ready-to-use therapeutic foods may be an alternative...’ [122]. The paper called for additional research on the use of enhanced family foods and cost comparisons to treatment with RUTF, but in the 10 years since this publication little research has followed in the area of family foods for treatment, in the midst of the expansion of RUTF and other ready-to-use foods. According to a recent Cochrane Review of interventions for treatment of MAM: ‘There are no studies evaluating interventions to improve the quality of the home diet, an approach that should be evaluated in settings where food is available, and nutritional education and habits are the main determinants of malnutrition’ [12]. The lack of research evaluating the effect of enhanced family foods for treatment should not be misunderstood as evidence for poor impact; rather, few have dared try this option in the wake of the ‘miracle food’ discovery and expansion of RUTF use. Furthermore, home-made enriched family foods are likely to have a positive influence on child feeding knowledge; serve as a good motivator for improved infant and young child feeding (IYCF) practice when a child is transformed with family food, and will build maternal self-confidence [122].

The 2007 Joint Statement on CMAM from the WHO, United Nations Standing Council on Nutrition, World Food Program and UNICEF stated that the community-based approach involved the provision of RUTFs or ‘other nutrient-dense foods at home’ for children suffering SAM without medical complications [8]. It seems that the latter option has been in many cases overlooked and rarely tried in favor of the ease and increased availability of the former. The use of homemade enriched family foods should work well for the rehabilitation phase but may require increased effort upfront in the realms of formative research, and delivery of effective counseling including necessary training. This approach would deliver long-lasting results that could not be achieved with the provision of RUTFs or any other ready-to-use food. Using local foods to increase nutrient intake among young children who are vulnerable to undernutrion has been shown to be acceptable and feasible in recent studies [123,124,125], and further research in this area for rehabilitation is warranted. Preparation of local foods may not be feasible in acute emergency situations when local, family foods truly are scarce. At such times, the benefits and ease of use of RUTFs may well be the best choice for treating children with SAM, but when this is the case and there truly are a lack of regular family foods there must be simultaneous actions taken to provide normal foods for the remaining population.

### 2.6. Cost and Unintended Use

There has been evidence of unintended use of RUTF by recipient families and staff at OTPs, affecting supply levels and raising sustainability concerns. The main challenges regarding RUTF misuse reported in UNICEF’s 2013 global evaluation of CMAM included sharing RUTF among siblings (without SAM), using it to treat MAM and families selling their RUTF supply for income [30]. A qualitative study of RUTF perceptions in Southern Ethiopia revealed that caregivers viewed RUTF as treatment for SAM but also as food for sharing with the family, and if necessary a product to be sold for household income [126].

Though the price has been declining [28], likely due to increased production capacity and demand [22,26], costs of RUTF remain exorbitant and unsustainable for routine health services in the poorest countries in Africa and Asia. Additionally, policy restrictions on bilateral funding frequently limit procurement to certain countries (e.g., made in the United States), further inhibiting the return or investment of funds to local economies. Currently the cost for an 8-week treatment of Plumpy’Nut for once child is around $50 [127]. The purchase price difference between imported versions (e.g., from Nutriset and Edesia) versus locally produced RUTF (e.g., Valid Nutrition, Malawi) is not large, with the local production costing a small amount more on average; in 2016 an 8-week treatment of RUTF for one child cost UNICEF $44.73 from Edesia in the US, $41.95 (€37.16) from Nutriset in France, and $59.24 from Valid Nutrition in Malawi [28]. The cost for locally produced RUTF has previously been cited as cheaper than the imported versions [122,128], and this likely varies considerably by location, production size, and other factors.

### 2.7. Alternatives

Alternative formulations of RUTF (mainly, alternatives to the peanut-base and/or milk powder) have been developed and tested in many different settings, both by private and public entities and partnerships. The search for formulas that can be made from products available locally and that are more cost-effective than the traditional peanut and milk powder based formulas is the driving force behind this. Most of these alternatives appear to be very similar to the original, peanut-based RUTF in packaging and use. Some contain rice, sesame, barley, lentils or soy, and many have removed milk powder altogether as a protein source in order to reduce costs.

The milk powder found in the traditional formula makes up about 30% of the final product but may account for more than half of the price, and even when made locally, the milk itself is often imported [24,129]. Bahwere et al. tested the effectiveness of using milk-whey protein in place of the milk powder in the traditional RUTF formula to treat Malawian children with SAM and found it to be a cheaper and effective alternative [129] but availability of whey varies widely. Weber et al. developed locally appropriate alternative formulas for the standard RUTF using a linear programming tool for Ethiopia, Ghana, Pakistan and India [130,131]. The tool optimized the formulas by identifying best ingredients to use based on nutrient needs, cost, and local availability. These alternatives were 60% cheaper than the traditional RUTF and were generally acceptable by mothers and children. Valid nutrition also developed a milk and peanut free version of the RUTF made from soya, maize, and sorghum to reduce costs of local production, and Bahwere et al. tested the product for efficacy against the peanut version in the Democratic Republic of Congo in 2011 [132]. Recovery was similar for children 24–59 months using the two products, but children ages 6–24 months did not do as well on the soy, maize, sorghum-RUTF compared to the peanut-RUTF [132]; the cheaper alternative may be viable for older children but more research is needed to understand the effect in younger children.

An additional alternative to RUTF and ready-to-use foods is blended foods, usually a combination of a starchy food (grains) and protein (beans/legumes), such as corn soy blend (CSB), or enriched/fortified blended foods, which add micronutrients to the blend. A Cochrane review conducted by Lazzerini et al. concluded that there was no difference in mortality, risk of default or progression to SAM in moderately malnourished children treated with lipid-based nutrient supplement (LNS)—products similar in form to RUTF—versus those treated with any blended foods, and although children treated with LNS had higher recovery rates the LNS also induced vomiting at a higher rate [12]. The authors also found that some enriched blended foods, such as a newly developed CSB++, performed equally as well as LNS and may be cheaper. Further, they identified one trial that found no significant benefit of the industrially produced CSB++ over locally made blended foods [133]. They highlighted the focus of studies testing industrially made products and found no studies that examined the impact of improving the home diet [12].

Regardless of the formula, location of production, ingredient origins, or cost, commercially processed foods are not ideally suited for tackling the nutritional problems of low-income populations around the world. In 1973 Popkin et al. [134] warned against the ‘dangers of commerciogenic nutritious foods’ and that the focus of using commercially processed nutritious foods for nutritional interventions could result in the deterioration of nutritional status amongst the poor rather than the intended improvement, as these products are likely replacing otherwise healthier, nutritionally adequate local foods, and cost more than real food alternatives. The term commerciogenic was first coined by Jelliffe [135] who sought to describe malnutrition in children ‘related to the unethical and inappropriate promotion of products by commercial infant food manufacturers…’ There is now once again, the potential to induce commerciogenic malnutrition with the advent of ready-to-use supplementary and weaning foods.

Even withholding projections of negative impact, the cost of RUTFs and other ready-to-use foods will remain an issue for the intended populations. The past experience of commercially processed supplementary foods can shed light on the potential trajectory of ready-to-use foods for malnutrition. For many years supplementary foods were commercially produced by nutrition researchers—like INCAPARINA, Faffa, UNIMIX—and these were acceptable to the targeted low-income user while the price was subsidized, but faded out once the subsidy disappeared, except maybe in middle class urban areas. The low-income mothers in low-income settings preferred the cheaper alternative of making meals themselves with foods they were used to preparing. These were the precursors of using RUTFs and ready-to-use supplementary foods (with lower fat content, and more preparation methods). The high costs of RUTFs and the expanding product line suggests they might face same fate once they are marketed at cost.

## 3. Conclusions

More information is needed on the cost effectiveness of enhanced family foods as an alternative to RUTFs. This is an urgent priority as: (1) this information is currently not available to nutrition policy makers and stakeholders who are driving demand for RUTF; and (2) it will vary according to several factors such as geographic location, season, and market conditions, among others.

Regardless of this, we argue that RUTFs, imported or locally manufactured, should only be used for children with SAM who have an appetite and are without medical complications, until they are discharged. After that they should transition to augmented local/family foods, and programs should include counseling and demonstrations on these. This will create a far more cost-efficient and sustainable solution, while empowering mothers with the skill and knowledge to care for her children in the future. Subjecting a mother to feed her child a prescription of commercially produced, medicalized food product, in the form of a single food type—sweetened, fatty peanut paste—for eight weeks cannot be lauded as nutritional best practice except for in the most dire of circumstances.

Nutrition programming must include integrated nutrition counseling, referral, and services where RUTFs are required (i.e., only in cases of SAM where family foods are impossible). International agencies must assess costs and opportunity costs associated not only with the use of RUTFs generally but also related to the displacement of resources from other long-term strategies for improving nutrition, e.g., nutrition sensitive agriculture and social and behavioral change. Care is needed to avoid RUTFs and new ready-to-use food products displacing appropriate complementary feeding and potential problems with using them for pregnant and lactating women.

In conclusion, the authors recommend a formal policy or code of practice regarding the RUTF industry and the use of such products. Several points should be considered. (1) Policy is needed to address the significant conflict of interest NGOs, like Valid, have in consulting on emergencies and promoting their own products. This is surely quite unethical; (2) As far as RUTF use is concerned, it should state that children, unless they are severely malnourished, should not get RUTFs. They should be better fed with their normal diet, while fostering improvements in IYCF (e.g., through community based nutrition programs); (3) Advertising and promotion of RUTFs should be regulated and subject to similar regulations as infant formula; (4) Finally, in emergencies, agencies should be obliged to avoid giving RUTFs to children other than those with current SAM unless they are really the only available foods; and if this must happen (i.e., there are no other foods available), this should trigger another emergency response in providing normal foods—staples, beans, oil, etc.

Vigilance is now needed to ensure that RUTFs and newly developed similar ready-to-use food products do not progress into the sphere of treating all nutrition problems resulting from food insecurity, beyond the scope of childhood SAM when rehabilitation with family foods is infeasible. The costs spent on research, development and procurement of additional RUTF and other ready-to-use foods, either for prevention, treatment or incorporation into the regular diet, are resources diverted from steps to address food insecurity at the root causes. Besides loss of resources for potentially better long-term investments, RUTF could be doing real harm by replacing healthy whole foods in the child’s diet with high-fat and high-sugar processed foods at a time when the double burden of under- and overnutrition is increasingly threatening the health of low and middle income country populations. Though there have been few studies to date, recent research has suggested potential negative long-term effects from SAM, evidenced by thrifty-growth, which has been shown associated with future chronic diseases [136]. Care should be taken to avoid leading children who have suffered SAM down a potential path towards obesity and chronic disease. In a 2015 commentary, Talukder stated this problem succinctly saying ”’magic’ solutions for childhood undernutrition prevention and treatment may have long term tragic consequences” [137].

Proponents of increasing the use of ready-to-use products for food security have promoted the notion that RUTFs are a “simple solution” to treating the global burden of malnutrition, yet long-term solutions need to be sustainable and emerge from within communities in need, not from a foil packet. The international nutrition community must continue to encourage nutritional best practices for children in all settings and push for long-term solutions to prevent food insecurity, both for chronic and emergency situations.
